# Novel Mutation Lys30Glu in the *TPM1* Gene Leads to Pediatric Left Ventricular Non-Compaction and Dilated Cardiomyopathy via Impairment of Structural and Functional Properties of Cardiac Tropomyosin

**DOI:** 10.3390/ijms252313059

**Published:** 2024-12-05

**Authors:** Elena V. Zaklyazminskaya, Victoria V. Nefedova, Natalia A. Koubassova, Natalia P. Kotlukova, Galina V. Kopylova, Anastasia M. Kochurova, Daniil V. Shchepkin, Natalia S. Ryabkova, Ivan A. Katrukha, Sergey Y. Kleymenov, Sergey Y. Bershitsky, Alexander M. Matyushenko, Andrey K. Tsaturyan, Dmitrii I. Levitsky

**Affiliations:** 1 Petrovsky National Research Centre of Surgery, Moscow 119991, Russia; helenezak@gmail.com; 2 Research Center of Biotechnology, A.N. Bach Institute of Biochemistry, Russian Academy of Sciences, Moscow 119071, Russia; victoria.v.nefedova@mail.ru (V.V.N.); s.yu.kleymenov@gmail.com (S.Y.K.); ammatyushenko@mail.ru (A.M.M.); 3 Institute of Mechanics, Lomonosov Moscow State University, Moscow 119192, Russia; natalia@imec.msu.ru; 4 Pediatrics Faculty of Medicine, Pirogov Russian National Research Medical University, Moscow 117997, Russia; natali130@yandex.ru; 5 Institute of Immunology and Physiology, Ural Branch of the Russian Academy of Sciences, Yekaterinburg 620049, Russia; g_rodionova@mail.ru (G.V.K.); d.shchepkin@iip.uran.ru (D.V.S.); serg.bersh@gmail.com (S.Y.B.); 6 Department of Biochemistry, Faculty of Biology, Lomonosov Moscow State University, Moscow 119234, Russia; n.ryabcova@gmail.com (N.S.R.);; 7 HyTest Ltd., 20520 Turku, Finland; 8 Koltzov Institute of Developmental Biology, Russian Academy of Sciences, Moscow 119334, Russia; 9 Department of Physiology and Pharmacology, Sackler Faculty of Medicine, Tel Aviv University, Tel Aviv 6997801, Israel; andrey.tsaturyan@gmail.com

**Keywords:** dilated cardiomyopathy, left ventricular non-compaction, tropomyosin, actin–myosin interaction, in vitro motility assay, molecular dynamics

## Abstract

Pediatric dilated cardiomyopathy (DCM) is a rare heart muscle disorder leading to the enlargement of all chambers and systolic dysfunction. We identified a novel de novo variant, c.88A>G (p.Lys30Glu, K30E), in the *TPM1* gene encoding the major cardiac muscle tropomyosin (Tpm) isoform, Tpm1.1. The variant was found in a proband with DCM and left ventricular non-compaction who progressed to terminal heart failure at the age of 3 years and 8 months. To study the properties of the mutant protein, we produced recombinant K30E Tpm and used various biochemical and biophysical methods to compare its properties with those of WT Tpm. The K30E substitution decreased the thermal stability of Tpm and its complex with actin and significantly reduced the sliding velocity of the regulated thin filaments over a surface covered by ovine cardiac myosin in an in vitro motility assay across the entire physiological range of Ca^2+^ concentration. Our molecular dynamics simulations suggest that the charge reversal of the 30th residue of Tpm alters the actin monomer to which it is bound. We hypothesize that this rearrangement of the actin–Tpm interaction may hinder the transition of a myosin head attached to a nearby actin from a weakly to a strongly bound, force-generating state, thereby reducing myocardial contractility. The impaired myosin interaction with regulated actin filaments and the decreased thermal stability of the actin–Tpm complex at a near physiological temperature likely contribute to the pathogenicity of the variant and its causative role in progressive DCM.

## 1. Introduction

Genetic dilated cardiomyopathy (DCM) is a rare progressive condition characterized by an enlarged left ventricular chamber volume, reduced systolic function, and impaired myocardial contractility [1,2]. In pediatric patients, the annual incidence rate is 0.34 to 3.8 per 100,000 in children under 18 years old, and 8.4 per 100,000 in infants (0–1 year) [3]. Although more than 100 genes are associated with DCM [4], even with extensive parallel sequencing, pathogenic variants are identified in only 30–40% of autosomal dominant forms and in 10–25% of sporadic DCM cases [5].

The *TPM1* gene (MIM*191010) encodes the major cardiac muscle isoform of tropomyosin (α-Tpm or Tpm1.1). Mutations in this gene are known to be causative for three main phenotypes—dilated cardiomyopathy (DCM, 1Y; MIM*611878), hypertrophic cardiomyopathy (HCM, 3; MIM*115196), and left ventricular non-compaction (LVNC, 9; MIM*611878) (https://omim.org/entry/191010, accessed on 15 October 2024). Cardiac remodeling caused by these mutations can be complex, with the primary phenotype of dilated or hypertrophic cardiomyopathy often accompanied by left ventricular non-compaction (LVNC) or restrictive features [4]. *TPM1*-related dilated cardiomyopathy accounts for about 1% of DCM cases [6].

Despite significant progress in medical treatment and improved survival (the annual death rate has decreased from 18% to 9%), pediatric DCM remains a significant cause of morbidity and mortality [7]. Genetic testing is crucial for risk evaluation, predictive testing of family members, and appropriate genetic counseling. A recent comprehensive review [7] highlights that pediatric DCM and LVNC can be associated with mutations in genes encoding both sarcomeric and non-sarcomeric proteins. To date, 54 single nucleotide variations (SNV) in the *TPM1* gene classified as Pathogenic or Likely Pathogenic are registered in the ClinVar database, and more than 400 rare variants remain categorized as variants of uncertain significance (VUS), awaiting functional characterization [8].

The Tpm1.1 isoform (hereafter referred to simply as Tpm) plays a key role, along with the troponin (Tn) complex, in the Ca^2+^-dependent regulation of contraction in cardiac and fast skeletal muscles [9,10]. The Tpm molecule is a typical α-helical coiled-coil dimer [10,11]. Due to the end-to-end interactions, Tpm dimers form continuous strands along the entire length of the actin filament [10,11]. In the absence of Ca^2+^, the Tpm–Tn complex sterically blocks the myosin-binding sites on actin, preventing myosin heads from interacting with actin. During muscle activation, Ca^2+^ binding to TnC leads the Tpm strand to move away from the blocking position, allowing myosin heads to bind to actin and generate force [9,10].

The structural and functional properties of this Tpm isoform can be significantly impaired by DCM-associated mutations in the *TPM1* gene. To understand the molecular mechanism by which these mutations lead to DCM development, it is necessary to know their effects on the Tpm properties. To date, there are about 30 mutations in the *TPM1* gene that have been identified to cause DCM [12]. However, the effects on the structure of the Tpm molecule and its functional properties have been studied for only a few of these mutations. Two of these muations, E40K and E54K, identified in the *TPM1* gene more than 20 years ago [13], have been the most intensively studied for their effects on Tpm properties. Numerous researchers have investigated these mutations using a variety of biochemical and biophysical methods [14,15,16,17,18,19,20]. Moreover, physiological effects of the E54K mutation have been studied in transgenic mice expressing the mutant Tpm in the adult heart [21].

The DCM-associated mutations studied to date have shown different effects on the Tpm affinity for F-actin: the E54K and K15N mutations decreased the affinity [15,22], while the D230N and A277V significantly increased it [23,24]. The E40K mutation did not affect the Tpm affinity for F-actin [15,19]. The effects demonstrated by the D84N mutation have been inconsistent: this mutation either decreased the affinity by 25% [25] or increased it [23].

At the same time, the DCM-associated mutations significantly impaired the regulatory properties of cardiac Tpm by reducing the Ca^2+^ sensitivity of thin filaments determined by different methods, such as the measurements of Ca^2+^-dependence of actin-activated ATPase activity of myosin [14,16,23,26,27] and sliding velocity of reconstructed thin filaments in the in vitro motility assay [14,18,19,24]. In particular, the DCM mutations E40K [14,18,19], D84N [23], D230N [23,26], T237S [27], and A277V [24] reduced the Ca^2+^ sensitivity by decreasing a *p*Ca_50_ value at which the half-maximal effect was achieved. Among the DCM-associated mutations studied, the effects of the E54K mutation obtained by different authors have been inconsistent: depending on the experimental conditions, this mutation either did not affect the *p*Ca_50_ value [17,18] or increased it [14,16].

It is important to note that cardiomyocytes from infants and young children with DCM were shown to demonstrate increased Ca^2+^ sensitivity compared with non-failing pediatric cardiomyocytes [28]. Based on these data, the authors suggested that contractile properties of pediatric DCM cardiomyocytes may differ significantly from those demonstrated in adult DCM carriers [28]. Although pediatric cardiomyopathies, including DCM, share many aspects with those in adults, there is growing evidence of their unique features that have implications for diagnosis and treatment [29]. It is also important to note that, due to a significant reduction in maximum force at saturating Ca^2+^ concentrations, the increased sensitivity did not result in a force increase at any physiological Ca^2+^ concentration [28].

In this study, we identified a novel de novo DCM-associated mutation, c.88A>G (p.Lys30Glu) in the *TPM1* gene, which has not been described before. This mutation (K30E) was found in a young girl who was diagnosed with DCM in her third week of life and who died at the age of 3 years and 8 months due to progressive heart failure (HF). The circumstances under which this genetic variant was discovered, along with the patient’s family history, did not allow us to obtain biological samples suitable for physiological experiments. Therefore, in order to evaluate the pathogenicity of this mutation, we produced recombinant K30E Tpm and applied various methods and approaches, such as differential scanning calorimetry (DSC), in vitro motility assay, and molecular dynamics (MD) simulations, to investigate its structural and functional properties. The results demonstrated that the K30E substitution significantly impairs the properties of Tpm, providing insights into the pathogenicity of this mutation.

## 2. Results

### 2.1. Clinical Case

The proband was born at the 40th week of a second normal pregnancy, through physiological delivery, weighing 3190 g and measuring 52 cm in length, with an Apgar score of 8/9. She had a febrile temperature up to 37.1 °C with normalization on the second day and was discharged from the hospital on the fifth day of life as a healthy baby. No concerns regarding cardiac complications in the fetus were raised. The mother noted that the baby was “perfectly calm, maybe too much”.

**Figure 1:**
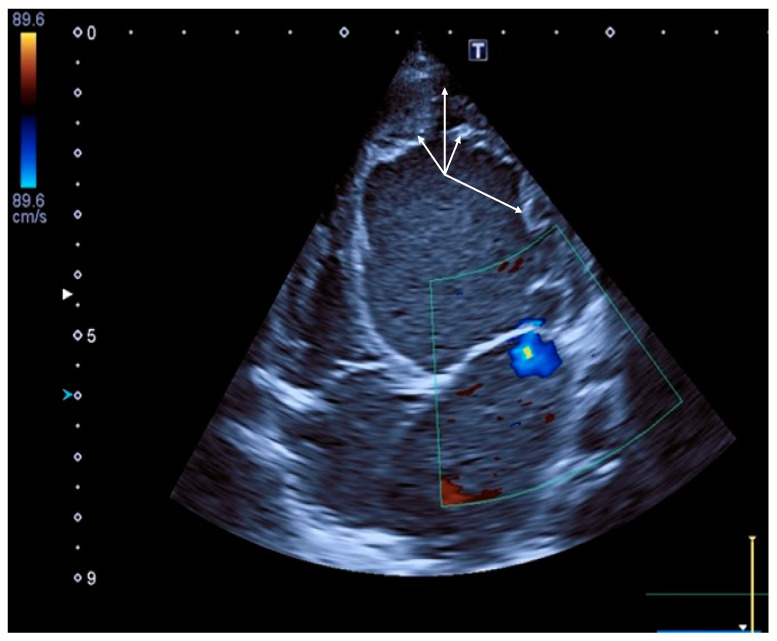
Fragment of the EchoCG of the proband (II.2) with dilated cardiomyopathy at the age of 1 year and 8 months. The left atrium (LA, bottom right) and left ventricle (LV, upper right) are shown. The end systolic size (ESS) is 36 mm (z-score 5.43), and the end diastolic size (EDS LV) is 43 mm (z-score 3.83). The left ventricular ejection fraction (LV EF) is 30%, and mitral regurgitation is at stage 2 (blue spot in the middle of the figure). High trabeculation and finger-like protrusion of the LV apex are seen on the top (indicated by arrows).

Two weeks later, she was hospitalized due to pallor, hyporeflexia, muscle hypotonia, diffuse cyanosis, and shortness of breath. Extensive clinical and instrumental investigations were performed. A cardiac ultrasound revealed LVNC, left and right ventricular dilatation, diffuse decreasing of contractility, and a left ventricular ejection fraction (LV EF) of 20% (Figure 1). After two weeks of medication, she was discharged from the pediatric hospital with LV EF 38–40% and clinical improvement. The patient was regularly followed up every 4–6 months and had appropriate updates on the treatment according to current guidelines. At the age of 2 years and 2 months, she suffered two consecutive respiratory infections complicated by acute bronchitis at home and was hospitalized at this age to the cardiac unit with circulatory failure. The dynamics of the main parameters of the cardiac ultrasound are summarized in Supplementary Table S1. During all this time she had cardiomyopathy with dilated phenotype, and decreased contractility, but stayed relatively asymptomatic and active despite failure to thrive.

During the entire observation period, the child received therapy for heart failure with regular dosage adjustment (digoxin, furosemide, spironolactone, captopril, cardiocytoprotective drugs, potassium and magnesium supplements).

Persistent clinical signs of progressive heart failure (weakness, shortness of breath, cyanosis) appeared at 3 years and 4 months. Her last hospitalization was at 3 years and 7 months when circulatory failure was complicated by acute respiratory infection. The patient died due to the progression of HF at the age of 3 years and 8 months.

### 2.2. Genetic Findings

**Figure 2:**
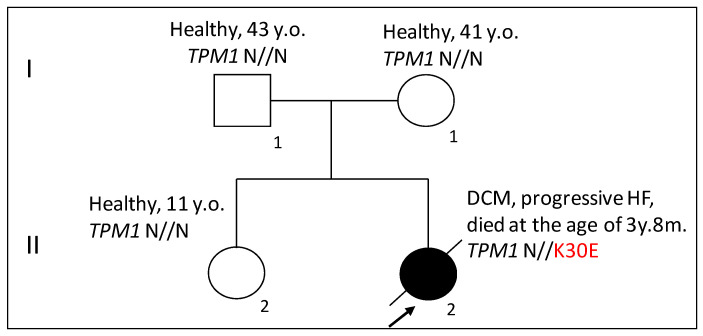
Pedigree of the DCM84 family. Healthy family members are shown with open symbols, and the affected proband is indicated with a closed symbol and an arrow. The abbreviations are given in the text. The generation number and individual numbers within the generation are marked by Roman and Arabic numerals, respectively. To evaluate the pathogenicity of the K30E mutation in the *TPM1* gene, we produced recombinant Tpm carrying the K30E substitution and applied various methods to investigate how this substitution affects the structural and functional properties of Tpm.

The family history was unremarkable, with no known cardiac disease in either parent, the older sister (10 years old), or other relatives (Figure 2). A genetic study using whole genome sequencing (WES) revealed a novel rare heterozygous genetic variant, hg19 chr15-63335116-A-G NM_001018005.2:c.88A>G (p.Lys30Glu, K30E) in the *TPM1* gene (see Supplementary Figure S1). No additional pathogenic or likely pathogenic variants, nor any unique variants of unknown clinical significance (VUS) were found in the scope of the bioinformatic analysis of >400 genes encoding sarcomeric and non-sarcomeric proteins known to be associated with inherited cardiomyopathies (dilated, hypertrophic, arrhythmogenic, and left ventricular non-compaction) (see Supplementary Table S2).

The K30E variant was not present in the older sibling or either parent, suggesting it arose de novo. The parents declined parenthood testing, so the PM6 criterion (PM6: assumed de novo, but without confirmation of paternity and maternity) was applied [30]. At the time of detection (2018), the variant was classified as a variant of uncertain significance (Class III, VUS) with criteria PM2, PM6, PP3 according to ACMG(2015) [30] and ClinGen Cardiomyopathy Expert Panel Specifications to the ACMG/AMP Variant Interpretation Guidelines for *TPM1* Version 1.0.0 (released 4/22/2024) (https://cspec.genome.network/cspec/ui/svi/affiliation/50002, accessed on 15 October 2024).

### 2.3. K30E Substitution Affects the Tpm Thermal Unfolding and Domain Structure Studied with Differential Scanning Calorimetry (DSC)

**Figure 3:**
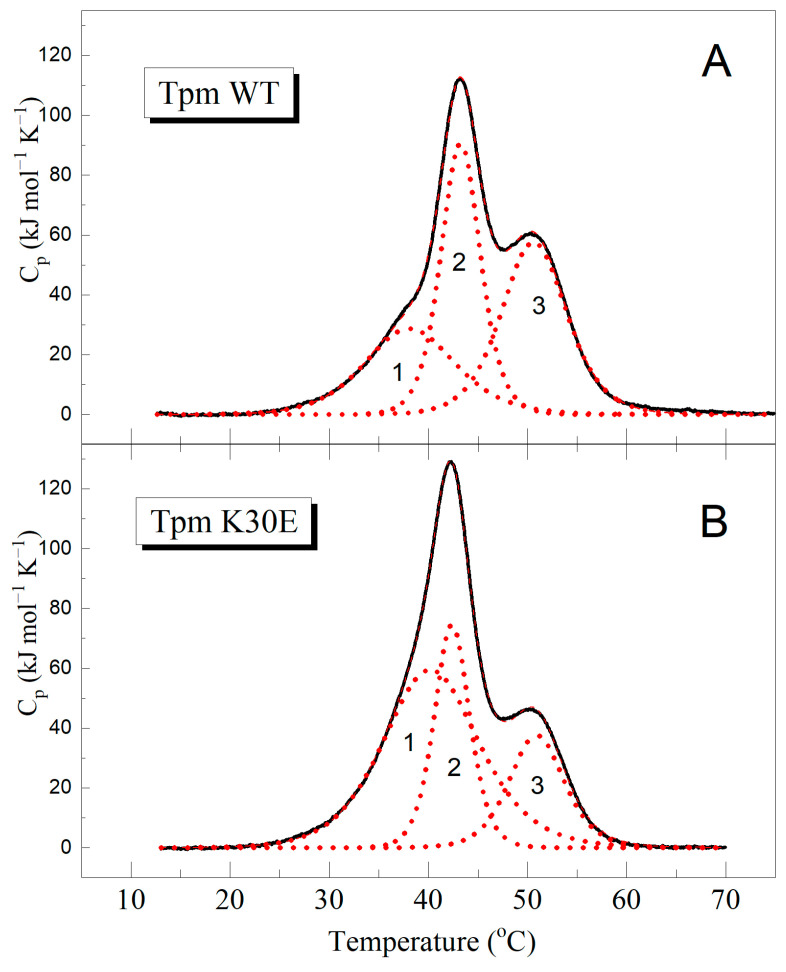
K30E substitution affects the structure of Tpm molecule. The plots show temperature dependences of excess heat capacity (C_p_) obtained from DSC studies on Tpm WT (**A**) and Tpm with the K30E substitution (**B**) along with their deconvolution into distinct thermal transitions (calorimetric domains 1, 2, and 3). The calorimetric parameters derived from the DSC data for individual thermal transitions are provided in Table 1.

The results obtained from DSC studies (Figure 3, Table 1) indicate that the K30E substitution causes significant structural changes in the Tpm molecule. Although this substitution had no appreciable effect on the maximum temperature (*T*_m_) of the three isolated thermal transitions (calorimetric domains) revealed upon thermal unfolding of the Tpm molecule, it led to significant decrease in the calorimetric enthalpy (the area under the thermal transition, Δ*H*_cal_) of domain 2 and especially domain 3, with corresponding increase, by more than twofold, in the enthalpy of the least thermostable calorimetric domain 1 (Figure 3, Table 1).

Previous studies assigned the calorimetric domains 2 and 3 of WT Tpm (Figure 3A) to the thermal unfolding of the C-terminal and N-terminal parts of Tpm, respectively [31,32]. In contrast, the least thermostable domain 1 was believed to reflect the unfolding of some other regions of the molecule, such as its middle part or the head-to-tail overlap junction between the N- and C-termini of neighbor Tpm molecules [32,33]. These DSC results indicated that the K30E substitution leads to serious destabilization of the N-terminal part of the molecule where it is located; as a result, some regions in this part of the molecule denature at much lower temperatures within the least thermostable calorimetric domain 1.

### 2.4. K30E Substitution Does Not Affect the Solution Viscosity of Tpm

The DSC results showing significant destabilization of some regions in the N-terminal part of the K30E Tpm molecule (Figure 3, Table 1) suggested that the K30E substitution, located rather close to the N-terminus, might affect the interaction between N- and C-ends of adjacent Tpm molecules. To test this assumption, we measured the solution viscosity of K30E Tpm in comparison with WT Tpm. The results showed that the viscosity of the K30E Tpm solution, after subtraction of the buffer viscosity, did not significantly differ from that for WT Tpm: the viscosity was equal to 0.28 ± 0.01 mPa·s for K30E Tpm vs. 0.32 ± 0.01 mPa·s for WT Tpm (mean ± SEM). This indicated that the K30E substitution has no appreciable effect on the interaction between the N- and C-ends of Tpm molecules.

### 2.5. K30E Substitution Only Slightly Increases the Tpm Affinity for F-Actin but Significantly Decreases the Thermal Stability of the Tpm–F-Actin Complex

**Figure 4:**
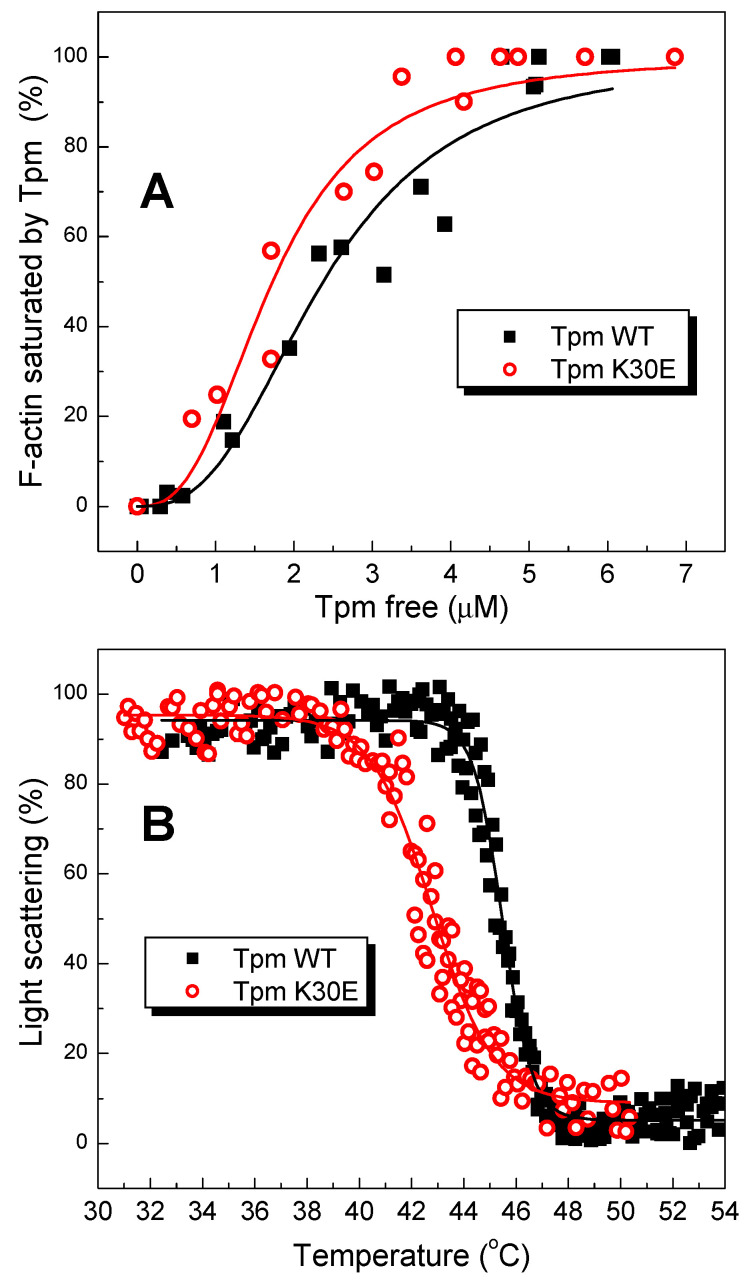
K30E substitution slightly enhances the actin-binding properties of Tpm but diminishes the thermal stability of the Tpm–F-actin complexes. The plots illustrate the effects of the K30E substitution on Tpm’s affinity for F-actin measured by the co-sedimentation assay in buffer containing 200 mM NaCl (**A**), and on the thermal stability of the Tpm–F-actin complexes, determined by light scattering measurements (**B**).

The effect of the K30E substitution on Tpm binding affinity for F-actin was measured using a co-sedimentation assay (Figure 4A). The results showed that this substitution slightly, but statistically insignificantly, increased the Tpm affinity for F-actin. The *K*_50%_ values (i.e., the Tpm concentration at which half of F-actin was saturated with Tpm molecules) were 2.45 ± 0.40 µM for Tpm WT and 1.87 ± 0.30 µM for Tpm K30E.

On the other hand, the K30E substitution significantly decreased the thermal stability of the Tpm*–*F-actin complex measured by the temperature-dependent decrease in light scattering, which reflects the dissociation of the complex (Figure 4B). The *T_diss_* values (i.e., the temperature at which a 50% decrease in the light scattering occurs) were equal to 42.70 ± 0.08 °C for Tpm K30E vs. 45.60 ± 0.03 °C for Tpm WT. It is important to note that dissociation of Tpm K30E from F-actin occurred within a broad temperature range (from 38.5 to 48.5 °C) which was much wider than that for Tpm WT, which dissociated within a narrower range from 43 to 48 °C (Figure 4B).

### 2.6. K30E Substitution Impairs Tpm Regulatory Properties

**Figure 5:**
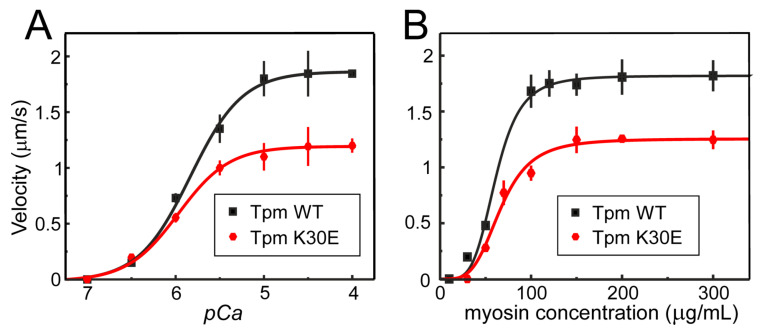
K30E substitution impairs the functional properties of Tpm. The plots display the effect of the K30E substitution in the Tpm molecule on the sliding velocity of regulated thin filaments along cardiac myosin in an in vitro motility assay depending on the Ca^2+^ concentration (*p*Ca) at a saturating myosin concentration equal to 300 µg/mL (**A**), and on the myosin concentration in the flow cell at a saturating Ca^2+^ concentration (at *p*Ca 4) (**B**). The experimental points represent the average values ± SD for the three experiments. Experimental data were fitted with the Hill equation. The sliding velocity parameters for WT Tpm and K30E Tpm derived from the data are presented in Table 2 and Table 3.

The K30E Tpm substitution resulted in approximately a one-third decrease in the maximal sliding velocity of the thin filaments at saturating Ca^2+^ concentration in the in vitro motility assay, compared to those containing WT Tpm (Figure 5A, Table 2). The substitution led to an increase in the Ca^2+^ sensitivity assessed by the *p*Ca_50_, from 5.83 ± 0.03 for WT Tpm to 5.97 ± 0.03 for K30E Tpm (Table 2).

Myosin–myosin cooperativity can be characterized by the dependence of thin filament velocity on myosin concentration in the in vitro motility assay [34,35]. Higher cooperativity indicates that fewer myosin molecules on the cover slip are required to move the filaments at maximal velocity. Although the K30E Tpm substitution decreased sliding velocity, neither the myosin concentration required to achieve a half-maximal velocity nor the Hill cooperativity coefficient changed (Figure 5B, Table 3).

### 2.7. Molecular Dynamics Simulation

To uncover the molecular mechanism behind the impaired Ca^2+^ regulation of cardiac thin filaments caused by the K30E substitution in Tpm, we performed molecular dynamics (MD) simulation of actin filaments complexed with WT Tpm or K30E Tpm. Additionally, we conducted an MD simulation of isolated Tpm dimers in solution. The latter simulation did not show any significant changes in bending stiffness or backbone h-bone occupancy. This result suggests that the primary reason for impairment is likely a modulation of the Tpm’s interaction with other partner proteins rather than changes in Tpm’s intrinsic properties.

**Figure 6:**
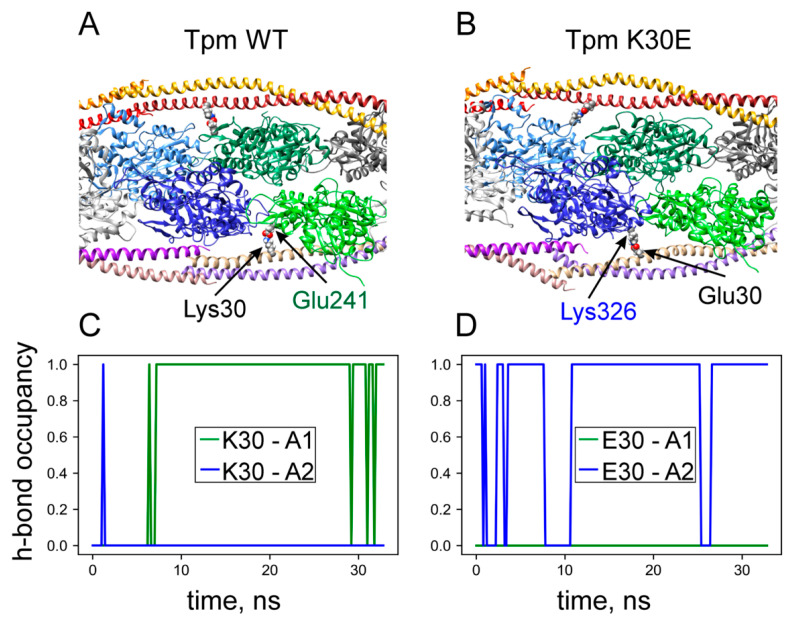
Top: snapshots of MD trajectories of actin–Tpm filaments with WT Tpm (**A**) and K30E Tpm (**B**). In the WT structure (**A**) Tpm residues Lys30 form hydrogen bonds with Glu241 residues of adjacent actin monomers, while in the K30E structure (**B**), the Glu30 residues of Tpm form hydrogen bonds with Lys326 of other actin monomers. The occupancies (1 indicates the presence; 0 indicates the absence) of hydrogen bonds between Tpm residue 30 and different actin monomers (A1, A2) for WT Tpm (**C**) and K30E Tpm (**D**). The green and blue actin monomers in **A** and **B** correspond to A1 and A2 in **C** and **D**, respectively, counting from the barbed end. Details of the MD simulations and the source of the initial atomic structure of the actin–Tpm complex are provided in Section 4.8.

Indeed, the MD simulation of the actin–Tpm filaments indicates that the K30E substitution alters the actin–Tpm interaction. The positively charged Lys30 of WT Tpm typically forms electrostatic and hydrogen bonds with the Glu241 of a neighboring actin monomer (Figure 6A,C). In contrast, the negatively charged Glu30 in the K30E Tpm forms stable bonds with positively charged Lys326 of another actin monomer (Figure 6B,D). Here and throughout, the numbering of actin residues corresponds to a 375-residue sequence (not the full 377-residue sequence), as the first two residues of α-actin are removed post-translationally [36,37].

Thus, the MD simulations suggest that the charge reversal of the 30th residue of Tpm due to the K30E substitution is accompanied by a change in the binding partner actin monomer. While the side chains of the Lys30 residue in WT Tpm and Glu241 residue of actin are aligned nearly perpendicularly to the thin filament axis (Figure 6C), the side chains of Glu30 in K30E Tpm and Lys326 residue of another actin monomer bound to it are tilted, suggesting the bond between them may cause a tensile force in the Tpm–Tn strands.

## 3. Discussion

### 3.1. Pathogenicity of the K30E Mutation in the TPM1 Gene

The primary objective of this research was to assess the pathogenicity of the novel de novo K30E mutation in the *TPM1* gene, identified in a proband with progressive HF. Clinical data indicated severe non-compaction and dilated cardiomyopathy (DCM) from the proband’s earliest days. To achieve our goal, we produced recombinant tropomyosin (Tpm) with the K30E substitution and investigated its structural and functional properties using various methods.

A significant finding was that the K30E Tpm substitution substantially impaired Ca^2+^ regulation of the actin–myosin interaction, markedly reducing the sliding velocity of the thin filaments across the entire range of physiological Ca^2+^ concentrations, particularly near or above its systolic value (Figure 5A, Table 2). The approximately 37% decrease in velocity suggests that the substitution significantly alters the Ca^2+^-dependent interaction of myosin heads with thin filaments in a manner characteristic of DCM.

The thermal destabilization of K30E Tpm and its complex with F-actin (Figure 3 and Figure 4B) may also play a role in the mutation’s pathogenicity. The temperature range at which the F-actin complex with the K30E Tpm becomes destabilized is closer to normal physiological conditions than initially thought. The temperature in a beating heart is better represented by the temperature in the pulmonary artery (PA) rather than in the axilla. PA temperature differs from axillary temperature by as much as 1.2 °C above or 1.6 °C below [38]. Although data on the physiological range of PA temperature in infants is limited due to the research challenges, at least one intensive care unit study found significant differences between intra-atrial and both axillary and rectal temperatures in children under 2 years old [39]. We hypothesize that the decreased stability of the F-actin complex with the K30E Tpm may contribute to the gradual deterioration of cardiac contractility in the proband, particularly during febrile episodes associated with respiratory or intestinal infections.

### 3.2. Possible Molecular Mechanism of the Pathogenicity of the K30E Tpm Substitution

A key finding from our in vitro motility assay experiments that shed light on the possible mechanism by which this substitution impairs heart function is the inhibition of actin–myosin interaction across the entire range of physiological Ca^2+^ concentration (Figure 5A, Table 2). The observed reduction in velocity could lead to a decrease in stroke volume and ejection fraction, ultimately causing myocardial remodeling and resulting in ventricular non-compaction and dilatation.

The motility assay data can be explained by a failure of myosin heads, weakly bound to some actin monomers, to transition into the strongly bound state necessary for producing mechanical force or filament sliding. Our MD simulation offers a plausible mechanism for how the K30E substitution rearranges the actin–Tpm interaction. The substitution of the positively charged Lys30 with negatively charged Glu30 disrupts the hydrogen and electrostatic bonds with the Glu241 residue of a neighboring actin monomer (Figure 6A,C). Instead, the Glu30 residue of mutant Tpm forms a bond with the Lys326 residue of the next actin monomer, closer to the pointed end of the actin filament (Figure 6B,D). We hypothesize that this replacement of the partner actin monomer at Tpm residue 30, caused by the K30E substitution, hampers the weak-to-strong binding transition of myosin head(s) bound to adjacent actin monomer(s). The force-generating transition is accompanied by a rotation of Tpm relative to the actin filament axis, likely breaking bonds between actin and Tpm [40,41]. An increase in the strength of the Tpm–actin bond due to the change in the actin binding partner at Tpm residue 30 could inhibit this weak-to-strong transition, reducing the number of force-generating myosin heads and consequently decreasing the sliding velocity of thin filaments in vitro (Figure 5A,B) and cardiac muscle force. The impairment in mechanical myocardial performance across the full range of physiological Ca^2+^ concentrations, along with reduced or nearly constant regulation cooperativity, is characteristic of DCM [42,43].

### 3.3. Comparison of the K30E Substitution with Other DCM-Associated Mutations in the TPM1 Gene

Our DSC results (Figure 3, Table 1) showed that the K30E substitution significantly destabilizes the Tpm molecule. In contrast, previous DSC studies indicated that various DCM mutations either had no appreciable effect on the Tpm thermal unfolding (E40K [15], M8R, and K15N [33]) or increased the thermal stability of the C-terminal (A277V [33]) or N-terminal (E54K [15]) regions of the molecule. Additionally, circular dichroism (CD) studies revealed that the DCM mutations D84N and D230N also increased the Tpm thermal stability at temperatures above 40 °C [23]. Therefore, the effects of the K30E on the thermal stability and domain structure of the Tpm molecule are quite different from those of other DCM mutations studied to date.

Our results showed that the K30E substitution did not significantly affect the viscosity of the Tpm solution, indicating that this substitution, located not far from the N-terminus of the Tpm molecule, has no appreciable effect on the interaction between the N- and C-termini of adjacent Tpm molecules. The head-to-tail interaction is believed to be the major determinant of Tpm solution viscosity [24,33]. In this regard, the effect of the K30E substitution is quite different from those of DCM mutations located close to the N- or C-termini of the molecule: mutations M8R and K15N in the N-terminal of Tpm caused a significant decrease in viscosity [33], whereas A277V mutation in the C-terminal of Tpm significantly increased it [24]. The difference can be explained by a recent high-resolution cryo-EM structural model of the Tpm junction region of the cardiac thin filament [44]. In contrast to Tpm residues M8, K15, and A277, which are involved in interaction with adjacent Tpm molecules at the overlap junction, residues 30 of both Tpm chains do not interact with either the adjacent Tpm molecule or the α-helical N-terminal fragment of TnT bound to the junction.

The K30E substitution caused only a slight increase in the Tpm affinity for F-actin, as measured by co-sedimentation assay (Figure 4A). However, it significantly decreased the thermal stability of the Tpm–F-actin complex (Figure 4B). This decrease can be explained by the fact that the stability of the Tpm–F-actin complexes is more dependent on the thermal stability of the Tpm molecule than on the Tpm’s affinity for F-actin [15,31,33].

The effect of the K30E substitution on the Ca^2+^ sensitivity of thin filament sliding velocity is notably different from that of other DCM-associated mutations, which either significantly decreased the Ca^2+^ sensitivity [14,16,18,23,24,26,27] or had no appreciable effect on it [17,18] (only in the case of E54K, but not E40K). It is worth mentioning that the effects of the E40K substitution on the Ca^2+^ sensitivity varied depending on the type of myosin used in the experiment: with ventricular myosin, this substitution considerably decreased the sensitivity, while with atrial myosin it did not affect the *p*Ca_50_ value [20]. Some other DCM-associated mutations, such as E54K [14,16,18], A277V [24], and T237S [27] had a minimal or no effect on the Ca^2+^-dependent activation of myosin interaction with thin filaments. Nevertheless, the sliding velocity of thin filaments with the K30E Tpm substitution was lower than that with WT Tpm across the entire range of physiological Ca^2+^ concentration. This suggests that the K30E Tpm substitution causes a significant impairment in myocardial contractility, which could, in turn, lead to cardiomyopathy.

### 3.4. Limitations

Our work focused on studying the properties of Tpm homodimers with the K30E substitution in both chains and comparing them with Tpm WT homodimers. However, the K30E mutation described here was heterozygous (see Section 2.2), indicating that the cardiac muscle of the young sudden cardiac death (SCD) victim contained a mix of WT Tpm, K30E Tpm, and WT/K30E dimers. Early studies demonstrated that equimolar mixtures of WT and mutant Tpm dimers had functional properties similar to those of mutant Tpm molecules [14]. The properties of Tpm dimers with the K30E substitution in only one chain may differ from those of the dimers with the substitutions in both chains. However, the molecular mechanism of impairing the actin–Tpm interaction proposed here suggests that at least half of the K30E/WT Tpm molecules form a bond with an actin monomer different from the one to which WT Tpm binds. Despite this limitation, the Tpm dimers with the K30E substitution in both chains were presumably present in the myocardium of the young SCD victim at a concentration sufficient to cause the structural and functional changes leading to the high pathogenicity of this mutation.

Our MD simulation was limited to actin–Tpm filaments without troponin due to the lack of a reliable, stable atomic model of the entire thin filament suitable for MD simulation. We were also unable to simulate the effect of the K30E Tpm substitution on the binding of myosin heads to a regulated thin filament. Although high-resolution structures of actin–Tpm filaments decorated with strongly bound myosin heads were recently obtained using cryo-EM [40,41], their atomic models were constructed using the helical reconstruction technique, which prevents reliable reconstruction of the side chains of protein residues. Despite the limited selection of the structural model for MD simulation, the change of the actin binding partner due to the K30E Tpm substitution predicted by our simulation appears quite plausible.

The clinical case described occurred several years ago, at a time when MRI was not routinely performed in children of such a young age. Additionally, no bacteriological or virological tests were conducted. Due to the absence of further investigations, alternative etiologies of DCM could not be excluded.

## 4. Materials and Methods

### 4.1. Clinical and Genetic Investigations

Clinical and genetic evaluations were performed following the Helsinki Declaration, its later amendments, and international clinical guidelines. Instrumental investigations included resting ECG and 24 h Holter ECG monitoring, Echo-CG (see Supplementary Figure S3). Genetic studies were performed based on the written informed consent of both parents of the proband and as a part of the clinical investigation. DNA was extracted from venous blood by standard reagent kits. Whole exome sequencing (WES) was performed on a NovaSeq 6000 instrument (Illumina platform, San Diego, CA, USA) with the SureSelect Human All Exon V7 (Agilent, Santa Clara, CA, USA) enrichment kit, following the manufacturer’s protocol. Familial screening was performed using capillary Sanger sequencing on an ABI 3500 Analyzer (ThermoFisher Scientific, Waltham, MA, USA). Reads were aligned to the human genome build GRCh37/UCSC hg19 and analyzed for sequence variants using a custom-developed bioinformatics pipeline. The pathogenicity of the rare variants was assessed based on ACMG (2015) recommendations [30]. The variant was registered in ClinVar, an open public database, with Variation ID: 3250489 and Accession Number VCV003250489.1.

### 4.2. Protein Preparations

Tpm species were recombinant proteins containing Ala-Ser N-terminal extension to mimic naturally occurring N-terminal acetylation of Tpm [45]. We used commercially available vector pet23a^+^. Tpm coding sequences were cloned between endonuclease restriction sites NdeI and EcoRI. The human K30E Tpm mutant was obtained by Q5 site-directed mutagenesis kit (NEB, NewEngland BioLabs, Ipswich, MA, USA). The following oligonucleotides were used:

5′-GACAAGGAGGCGGCGGAAGAC-3′ as a forward primer (mutant codon is underlined) and 5′-GGCCTCCGCCTGCTCAGCTC-3′ as an adjacent primer. The resulting CDS was verified by sequencing. Tpm proteins were obtained and purified as previously described [33,46]. The purity of Tpm species was >95% (see Supplementary Figure S2). The Tpm concentrations were determined spectrophotometrically at 280 nm using an *E*^1%^ of 2.7 cm^−1^.

Myosin, extracted from the left ventricle of the ovine heart, and actin from *m. psoas* of a rabbit were prepared using standard methods [47,48]. In some cases, filamentous actin (F-actin) polymerized from monomeric G-actin was further stabilized with phalloidin or labeled with TRITC-phalloidin (Sigma Chemical Co., St. Louis, MO, USA), as previously described [33,46]. A recombinant human cardiac troponin (Tn) complex was provided by HyTest (Turku, Finland, Cat.# 8ITCR). The purity of the Tn complex composed of TnT, TnI, and TnC was >95% (see Supplementary Figure S2).

All procedures involving animal care and handling were performed according to institutional guidelines set forth by the Animal Care and Use Committee at the Institute of Immunology and Physiology Ural Branch of RAS, and Directive 2010/63/EU of the European Parliament.

### 4.3. Differential Scanning Calorimetry (DSC)

DSC experiments were conducted as described earlier [33,46,49] on a MicroCal VP-Capillary DSC differential scanning calorimeter (Malvern Instruments, Northampton, MA, USA) at a heating rate of 1 °C/min and a protein concentration of 2 mg/mL in 30 mM Hepes-Na buffer (pH 7.3) containing 100 mM NaCl. Before DSC experiments, Tpm species were reduced by heating at 60 °C for 20 min in the presence of 3 mM DTT [33,46,49]. The thermal unfolding of Tpm was fully reversible. The temperature dependence of the excess heat capacity was analyzed and plotted using Origin 7.5 software (MicroCal Inc., Northampton, MA, USA). Deconvolution analysis of the heat sorption curves, i.e., their decomposition into separate thermal transitions (calorimetric domains) was performed as previously described [31,32,33,46].

### 4.4. Viscosimetry

The viscosity measurements of the Tpm solutions were performed using a falling ball micro viscometer Anton Paar AMVn (Ashland, VA, USA) in a 0.5 mL capillary at 20 °C, as previously described [33,46,49]. All measurements were performed at a Tpm concentration of 2.0 mg/mL in a 30 mM Hepes-Na buffer (pH 7.3) containing 100 mM NaCl and 4 mM DTT. The viscosity of each Tpm sample was measured at least three times, and the viscosity values over buffer viscosity were averaged.

### 4.5. Cosedimentation of Tpm Species with F-Actin

The apparent affinity of Tpm species for F-actin was estimated using a cosedimentation assay [33,49,50]. Briefly, 10 µM F-actin was mixed with increasing concentrations of Tpm at 20 °C in 30 mM Hepes-Na buffer (pH 7.3) containing 200 mM NaCl. Actin was pelleted by ultracentrifugation at 100,000× *g* for 40 min, and equivalent samples of the pellet and the supernatant were subjected to SDS-PAGE [51]. Protein bands were scanned and analyzed using ImageJ 1.53k software (Scion Corp., Frederick, MD, USA). Three measurements were performed for each Tpm sample.

### 4.6. Temperature Dependences of Light Scattering

The thermally induced dissociation of the Tpm complexes with F-actin, stabilized by phalloidin, was measured by changes in light scattering intensity at a 90 degree angle, as previously described [33,49,50]. The experiments were conducted at a 350 nm wavelength and a constant heating rate of 1 °C/min on a Cary Eclipse fluorescence spectrophotometer (Varian Australia Pty Ltd., Mulgrave, Australia). The solutions contained 20 µM F-actin and 10.5 µM Tpm in 30 mM Hepes-Na buffer (pH 7.3) with 100 mM NaCl. The dissociation curves were analyzed by fitting to a Boltzmann sigmoidal decay function. The main parameter extracted from this analysis was *T*_diss_, i.e.*,* the temperature at which a 50% decrease in the light scattering occurs.

### 4.7. In Vitro Motility Assay

Measurements of the Ca^2+^-dependent sliding velocities of regulated thin filaments were performed in the in vitro motility assay at 30 °C, as previously described [49,52]. Regulated thin filaments were reconstructed by adding 100 nM Tpm and 50 nM Tn complex to 10 nM TRITC-phalloidin–labeled F-actin and placed into an experimental flow cell with ovine cardiac myosin immobilized on the nitrocellulose-coated inner surface. The sliding velocity was analyzed with GMimPro2023 software [53]. Each Tpm sample was tested in three independent experiments with newly prepared myosin. All values are expressed as mean ± SD. Comparisons were performed by paired *t*-test or Mann-Whitey *U* test at a significance level of 0.05 (*p* < 0.05).

To investigate the effect of the K30E Tpm substitution on the cooperativity of myosin interaction with regulated thin filaments, the dependence of the filament sliding velocity on the surface density of myosin in the flow cell, varied by infusion of different myosin concentrations in the cell, was analyzed as described [34,35,49,52].

### 4.8. Molecular Dynamics (MD)

Our MD modeling was based on the refined thin filament structural model in the Ca-bound state (pdb ID 7UTI, [54]). Troponin complexes were removed due to their instability in short MD runs. The unit, containing 16 actin monomers with two Tpm strands, was aligned so that the actin principal axis coincided with the *z*-axis using the orient scripts in VMD [55]. The extended actin–Tpm construct was built according to the procedure described in [54], but with an axial length of 26 actin monomers, as in our previous Ca-free state actin–Tpm model [56]. The 30th Tpm residue of interest was positioned in the central part of the model to prevent higher fluctuations of the free ends from affecting its interactions with actin. The Tpm K30E substitutions were introduced using a rotamer library [57] in UCSF Chimera [58].

The calculations were performed in GROMACS v. 2021.5 [59] as was described in [56] using the water model TIP3P and the CHARMM36 force field [60]. The length of MD trajectories was 33 ns for both models (with WT Tpm and Tpm K30E). The figure was rendered with UCSF Chimera [58].

## 5. Conclusions

A multifaceted study was conducted to analyze the functional consequences of K30E substitution in the Tpm protein and to elucidate its role in pathogenesis of progressive DCM. The observed changes in protein properties are consistent with the clinical phenotype of the proband, indicating a reduction in muscular contraction efficiency. Based on the data presented, we propose that the c.88A>G (K30E) variant is a novel pathogenic mutation that arose de novo and is associated with a rapidly progressive course of disease. Additionally, we suggest that core body temperature and febrile episodes may be underestimated yet potentially controllable factors in modulating certain genetically determined HF conditions, particularly those involving a decrease in the thermal stability of sarcomeric proteins.

## Figures and Tables

**Table 1:** Calorimetric parameters obtained from the DSC data (Figure 3) for separate thermal transitions (calorimetric domains) of WT Tpm and Tpm with mutation K30E.

Tpm	*T*_m_ ^a^ (°C)	Δ*H*_cal_ (kJ mol^−1^)	Δ*H*_cal_ (% of Total)	Total Δ*H*_cal_ ^b^ (kJ mol^−1^)
**Tpm WT**				1330
Domain 1	38.2	350	26	
Domain 2	43.3	480	36	
Domain 3	50.7	500	38	
**Tpm K30E**				1390
Domain 1	40.3	760	55	
Domain 2	42.4	355	25	
Domain 3	50.9	275	20	

^a^ The error of the given values of transition temperature (*T*_m_) did not exceed ±0.2 °C. ^b^ The relative error of the given values of calorimetric enthalpy, Δ*H*_cal_, did not exceed ±10%.

**Table 2:** Characteristics of the *p*Ca-velocity relationship of thin filament containing WT Tpm or K30E Tpm in the in vitro motility assay.

Tpm	V_max_ (µm/s)	V_0_ (µm/s)	*p*Ca_50_	*n*
WT Tpm	1.86 ± 0.03	0	5.83 ± 0.03	1.47 ± 0.1
K30E Tpm	1.18 ± 0.02 **	0	5.97 ± 0.03 *	1.50 ± 0.12

V_max_, the maximum sliding velocity of thin filaments at saturating Ca^2+^ concentration; V_0_, the sliding velocity at *p*Ca 7.5; *p*Ca_50_ is the *p*Ca value at which the sliding velocity is half-maximal; *n*, Hill cooperativity coefficient. Statistically significant differences in characteristics of Tpm with K30E substitution from those of WT Tpm, *p* < 0.05 and *p* < 0.01, are marked by symbols (*) and (**), respectively.

**Table 3:** Characteristics of myosin concentration dependence of sliding velocity of thin filament containing WT Tpm or K30E Tpm in the in vitro motility assay.

Tpm	V_max_ (µm/s)	[myo]_50_ (µg/mL)	*n*
WT Tpm	1.84 ± 0.04	61.3 ± 2.8	4.49 ± 0.66
K30E Tpm	1.27 ± 0.05 *	66.4 ± 3.5	3.95 ± 0.76

V_max_, the maximum sliding velocity of thin filaments at saturating myosin concentration; [myo]_50_, myosin concentration at which the sliding velocity is half-maximal; *n*, Hill cooperativity coefficient. Statistically significant difference in characteristics of Tpm with K30E substitution from those of WT Tpm, *p* < 0.01 is marked by symbol (*).

## Data Availability

All information related to the patient/family is completely anonymized and unidentifiable. The raw clinical data supporting this article cannot be placed in a public repository due to ethical reasons (personal data protection) but it will be available from the co-author (E.V.Z., zhelene@mail.ru) upon reasonable request. All experimental data generated or analyzed during this study are included in this article. The genetic data was registered in open public database ClinVar, with Variation ID: 3250489 and Accession Number VCV003250489.1.

## Supplementary Materials

The following supporting information can be downloaded at: https://www.mdpi.com/article/10.3390/ijms252313059/s1.
