# Supplementary material for: Novel Mutation Lys30Glu in the TPM1 Gene Leads to Pediatric Left Ventricular Non-Compaction and Dilated Cardiomyopathy via Impairment of Structural and Functional Properties of Cardiac Tropomyosin
Source: Int J Mol Sci. 2024 Dec 5;25(23):13059. doi: 10.3390/ijms252313059 (PMC11641563; doi:10.3390/ijms252313059)
Supplement: Supplementary file 1 [file ijms-25-13059-s001.zip › ijms-3318526-supplementary.pdf]

<u>Supplementary Data</u>

to the manuscript "*De novo* mutation Lys30Glu in the *TPM1* gene leads to pediatric left ventricular non-compaction and dilated cardiomyopathy via impairment of structural and functional properties of cardiac tropomyosin"

by E.V. Zaklyazminskaya, V.V. Nefedova, N.A. Koubassova, N.P. Kotlukova, G.V. Kopylova, A.M. Kochurova, D.V. Shchepkin, N.S. Ryabkova, I.A. Katrukha, S.Y. Kleymenov, S.Y. Bershitsky, A.M. Matyushenko, A.K. Tsaturyan, and Dmitrii I. Levitsky

submitted to International Journal of Molecular Sciences (Special Issue "Research Progress on the Mechanism and Treatment of Cardiomyopathy").

Table S1.
Parameters of proband's echocardiography registered at different ages.

**Age**	**1 day**	**3 weeks**	**2 months**	**3 months**	**7 months**	**1 year**	**2 years 3 months**	**3 years 4 months**	**3 years 7 months**
Weight, kg	3.190	3.290	3.888	4.560	n.d.	7.500	10.5	11.8	11.9
Height, cm	53	53	n.d.	59	n.d.	n.d.	88	93	95
BMI	11.4	11.7	n.d.	13.1	n.d.	n.d.	13.6	13.64	13.2
LVEDD, mm	n.d.	21.7	n.d.	26	3	36	43	42	44
LVESD, mm	n.d.	1.81	n.d.	n.d.	n.d.	n.d.	3.6	36.4	36.7
LV EF (%)	n.d.	37	50	40	42-46	36-43	34	34-36	35
FS (%)	n.d.	16	n.d.	n.d.	n.d.	n.d.	16	15-17	16-18
Mitral regurgitation	0	1,5+	n.d.	1+	1+	n.d.	2+	2+	2+
Pulmonary artery	n.d.	Normal	Normal	Normal	Normal	n.d.	dilated, pulmonary regurgitation, 1.5+	dilated, pulmonary regurgitation, 1.5+	dilated, pulmonary regurgitation, 1.5+
NC/C ratio	1.48	1.48	1.48	1.48	1.48	1.48	1.48	1.48	1.48

Table S1 (continued)									
LV anatomy	n.d.	Foramen ovale, patent ductus arteriosis. Increased trabeculation in the medium and apical segments of the LV.	n.d.	n.d.	n.d.	Increased trabeculation in the medium and apical segments of the LV, finger-like aneurism of the apical segment of the LV.	Increased trabeculation in the medium and apical segments of the LV, finger-like aneurism of the apical segment of the LV, trabeculae are located transversely, giving the left ventricle an S-shape.	yes	yes
Hypokinesis of the IVS	no	n.d.	yes	yes	n.d.	n.d.	yes	yes	yes
Pulmonary hypertension, mm Hg	no	no	n.d.	n.d.	yes	yes	35	38-40	48
Tricuspid regurgitation	no	n.d.	n.d.	n.d.	2+	1.5-2+	1+	1+	1+
Diastolic dysfunction	no	n.d.	n.d.	n.d.	n.d.	n.d.	E>A	E/A 2.4	E/A 2.4
RA, mm	normal	n.d.	n.d.	n.d.	n.d.	18	n.d.	24	28
LA, mm	normal	n.d.	n.d.	n.d.	n.d.	27	n.d.	n.d	25.5
IVS diastolic	normal	n.d.	n.d.	n.d.	3.5	n.d.	n.d.	4.5	4.7

Abbreviations: A - late diastolic transmitral flow velocity; BMI - body mass index; E - peak early diastolic flow velocity; FS - fractional shortening; IVS - interventricular septum; LVEDD - Left ventricular end diastolic diameter; LVESD - Left ventricular end systolic diameter; LV EF - left ventricular ejection; NC/C - non compact/compact layer of myocardium; RA - right atrium fraction; LA - left atrium fraction.

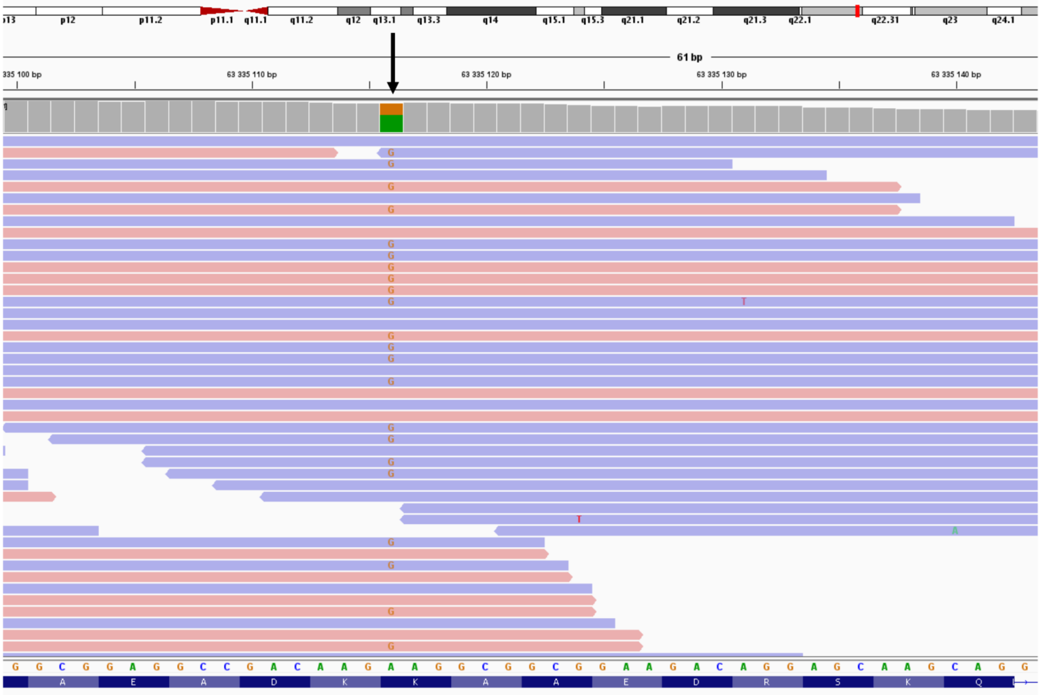

Figure S1. Fragment of the whole exome sequencing (WES) of the proband’s DNA sample. Data visualization performed in the Integrative Genome Viewer (IGV) 2.17.2 using hg19/GRChr37 as a reference genome. Blue and pink lines correspond to the direct and reverse reads. Heterozygous variant TPM1(NM_001018005.2):c.88A>G p.(Lys30Glu) is marked by arrow with a total number of 108 reads (65 reads per reference allele; 43 reads per alternative allele).

Table S2.
List of genes encoding sarcomeric and non-sarcomeric proteins, which were analyzed for the DCM proband.

AARS2, ABCC6, ABCC9, ABL1, ACAD9, ACADVL, ACTA1, ACTA2, ACTB, ACTC1, ACTG1, ACTN2, ACVR1, ACVR2B, ACVRL1, ADAMTS10, ADAMTS17, ADAMTS2, AFF4, AGK, AGL, AGPAT2, AKAP9, ALMS1, ALPK3, AMMECR1, ANK2, ANO5, APOA1, ARHGAP31, ARID1A, ARID1B, ATPAF2, B3GAT3, B3GLCT (B3GALTL), BAG3, BAG5, BBS2, BCOR, BMPR2, BRAF, C12orf57, CACNA1C, CACNA1D, CACNA2D1, CACNB2, CALM1, CALM2, CALM3, CALR3, CAPN3, CASQ2, CASZ1, CAV3, CBL, CBS, CCDC39, CCDC40, CDH2, CDK13, CDK9, CFAP45, CFAP52, CFAP53, CFC1, CHD4, CHD7, CHRM2, CHST14, CITED2, COL1A1, COL1A2, COL3A1, COL5A1, COL5A2, COX15, CPT2, CREBBP, CRELD1, CRPPA (ISPD), CRYAB, CSRP3, CTC1, CTNNA3, DBH, DCHS1, DES, DHCR7, DLL4, DMD, DNAAF1, DNAH5, DNAI1, DNAJC19, DOCK6, DOLK, DPM3, DPP6, DSC2, DSG2, DSP, DTNA, DYSF, EEF1A2, EFTUD2, EHMT1, EIF2AK4, ELAC2, ELN, EMD, ENG, ENPP1, EOGT, EP300, EPG5, EPHB4, ESCO2, ETFA, ETFB, ETFDH, ETS1, EVC, EVC2, EYA4, F2, F5, FAH, FARS2, FBN1, FBN2, FBXL4, FBXO32, FGF12, FHL1, FHOD3, FKBP14, FKRP, FKTN, FLNA, FLNC, FLT4, FOXC1, FOXC2, FOXD4, FOXF1, FOXH1, FOXP1, FOXRED1, FXN, G6PC3, GAA, GATA4, GATA5, GATA6, GATAD1, GATC, GBE1, GDF1, GDF2, GFM1, GJA1, GJA5, GLA, GLB1, GMPPB, GNAI2, GNB2, GNB5, GPC3, GPD1L, GSK3B, GSTM3, GTPBP3, GUSB, HADHA, HAND1, HAND2, HCN2, HCN4, HDAC8, HEY2, HFE, HNRNPK, HOXA1, HRAS, IDUA, ILK, JAG1, JPH2, JUP, KANSL1, KAT6A, KAT6B, KCNA5, KCNAB2, KCNB2, KCND2, KCND3, KCNE1, KCNE2, KCNE3, KCNE5, KCNH2, KCNJ16, KCNJ2, KCNJ5, KCNJ8, KCNQ1, KCNT1, KDM6A, KLHL24, KMT2D, KRAS, KYNU, LAMA2, LAMA4, LAMP2, LARGE1, LDB3, LEFTY2, LEMD2, LMNA, LMOD2, LOX, LRRC10, LZTR1, MAP2K1, MAP2K2, MAP3K8, MAPK1, MED12, MED13L, MEIS2, MFAP5, MIB1, MIPEP, MKKS (BBS6), MKS1, MLYCD, MMP21, MNS1, MRAS, MRPL3, MRPL44, MRPS22, MTO1, MYBPC3, MYBPHL, MYCN, MYH6, MYH7, MYL2, MYL3, MYL4, MYLK2, MYLK3, MYO18B, MYOT, MYOZ2, MYPN, MYRF, NAA10, NAA15, NDUFAF2, NEXN, NF1, NFATC1, NIPBL, NKX2-5, NKX2-6, NODAL, NONO, NOS1AP, NOTCH1, NOTCH2, NPHP3, NPPA, NR2F2, NRAP, NRAS, NSD1, NUP155, OFD1, PARS2, PBX1, PCCA, PCCB, PITX2, PKD1, PKD1L1, PKP2, PLD1, PLEC, PLEKHM2, PLN, PNPLA2, POMT1, PPA2, PPCS, PPP1CB, PPP1R13L, PRDM16, PRDM6, PRKAG2, PRKD1, PRKG1, PSEN1, PSEN2, PTPN11, PUF60, QRSL1, RAB23, RAF1, RAI1, RANGRF, RASA1, RASA2, RBCK1, RBM10, RBM20, RECQL4, RERE, RIT1, RMND1, ROR2, RPL3L, RPL5, RPS19, RRAS, RRAS2, RYR2, SALL1, SALL4, SCN10A, SCN1B, SCN2B, SCN3B, SCN4B, SCN5A, SCNN1A, SCNN1B, SCNN1G, SCO1, SCO2, SDHA, SELENON, SEMA3A, SGCA, SGCB, SGCD, SGCG, SHOC2, SKI, SLC22A5, SLC25A20, SLC25A3, SLC25A4, SLC2A10, SLC39A13, SLC4A3, SLMAP, SMAD2, SMAD3, SMAD4, SMAD6, SMARCA4, SMARCB1, SMC1A, SMC3, SMCHD1, SNTA1, SON, SOS1, SOS2, SPEG, SPRED1, SPRED2, STAG2, STRA6, TAB2, TANGO2, TAZ, TBX1, TBX20, TBX5, TCAP, TECRL, TFAP2B, TGDS, TGFB2, TGFB3, TGFBR1, TGFBR2, TJP1, TKT, TLL1, TMEM168, TMEM43, TMEM70, TMEM94, TNNC1, TNNI3, TNNI3K, TNNT2, TNXB, TOR1AIP1, TP63, TPM1, TRDN, TRIM32, TRPM4, TSFM, TTN, TTR, UBR1, VARS2, VCL, VCP, VPS13A, WSCD1, XIRP1, XIRP2, XK, ZEB2, ZFPM2, ZIC3.

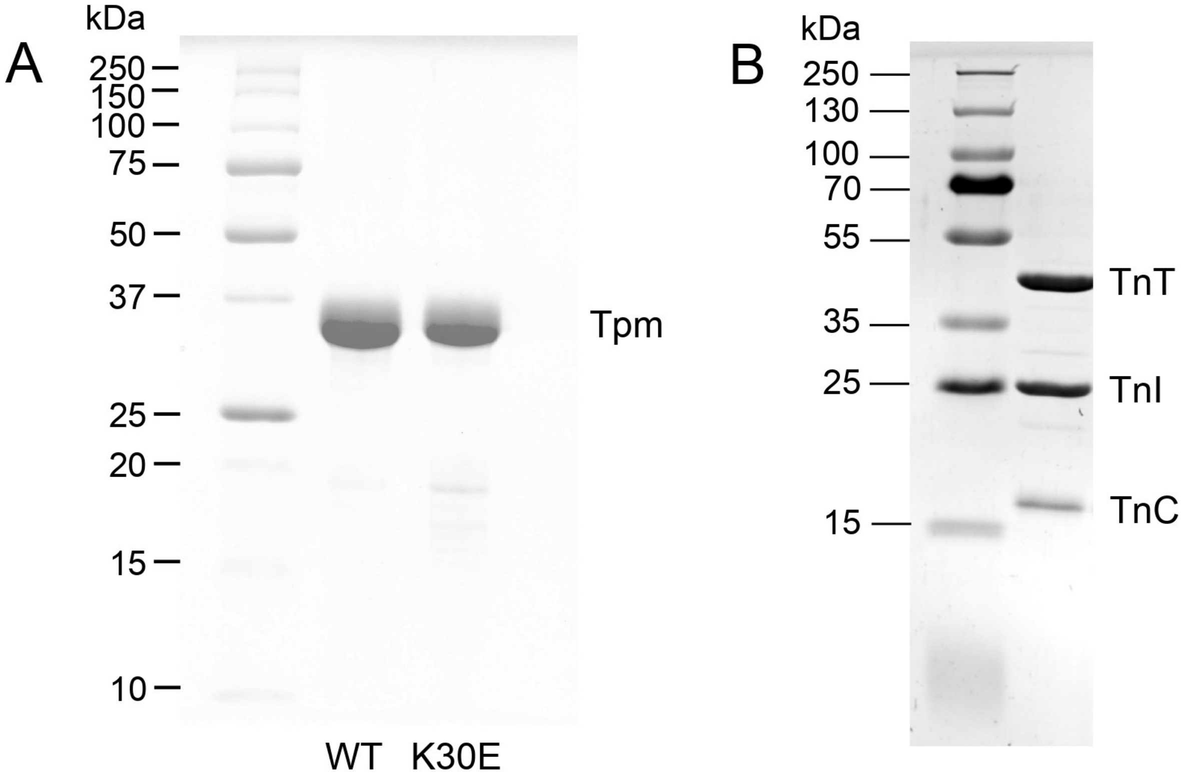

Figure S2. SDS-PAGE electrophoregrams of recombinant proteins used in the study: **A** - Tropomyosin species (Tpm WT and Tpm K30E); **B** - Troponin complex composed of TnT, TnI, and TnC. The purity of Tpm and Tn was > 95%.

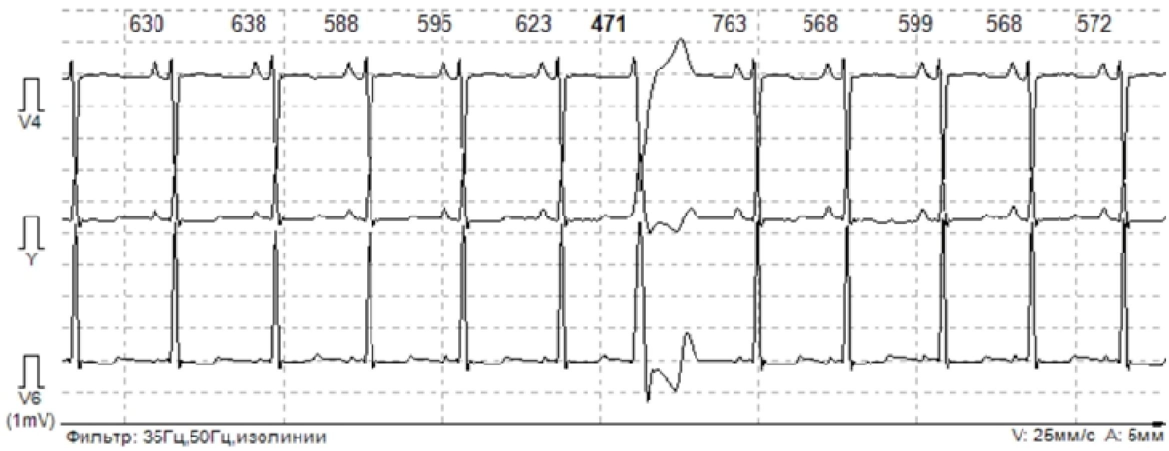

Figure S3. Fragment of the ECG Holter monitoring (22 h 10 min) registered at 3 y. 4 m. (3 month before death due to progression of the heart failure). Lone ventricular extrasystole.

General characteristics: HR 81-167 bpm, sinus arrhythmia with a tendency to sinus tachycardia, circadian index 1,21 (rigid rhythm), maximal pause 1047 ms, Minimal QTc 380 ms, maximal 471 ms, maximal PQ 130 ms. Ectopic activity during 24 hour: 2 ventricular extrasystolies, 2 supra-ventricular extrasystolies**.**
